# Real-World Evidence on the Prevalence of Molar Incisor Hypomineralization in School Children from Bucharest, Romania

**DOI:** 10.3390/children10091563

**Published:** 2023-09-16

**Authors:** Beatrice Ciocan, Mihai Săndulescu, Rodica Luca

**Affiliations:** 1 Doctoral School, Faculty of Dentistry, Carol Davila University of Medicine and Pharmacy, 17-23 Calea Plevnei, 010221 Bucharest, Romania; 2 Department of Implant-Prosthetic Therapy, Faculty of Dentistry, Carol Davila University of Medicine and Pharmacy, 17-23 Calea Plevnei, 010221 Bucharest, Romania; 3 Department of Pedodontics, Faculty of Dentistry, Carol Davila University of Medicine and Pharmacy, 17-23 Calea Plevnei, 010221 Bucharest, Romania

**Keywords:** MIH, dental hypomineralization, pedodontics, screening

## Abstract

Molar incisor hypomineralization (MIH) is an understudied and underrecognized clinical entity occurring in children. We performed a cross-sectional study to determine the real-world prevalence of MIH among school children undergoing routine dental checkups at one primary and middle school in Bucharest, Romania. Our study cohort consisted of 266 children with evaluable data, of which 143 (53.8%) were males, with a median age of 10 years old (interquartile range: 8–11 years). In this study cohort, we have identified a prevalence of 14.3% (*n* = 38 cases) of MIH. Among patients diagnosed with MIH, hypomineralizations were present in 47.4% of children on the maxillary first molar, 92.1% on the mandibular first molar, 94.7% on the maxillary incisor, 36.8% on the mandibular incisor, and 5.3% on the deciduous second molar. We identified the maxillary incisor and the mandibular first molar as the most important examined sites significantly associated with the presence of MIH (*p* < 0.0001 each), highlighting the importance of paying focused attention to these sites during routine dental care in children. In order to establish the diagnosis of MIH, findings of hypomineralization should be present on at least one permanent first molar, according to the case definition currently in use; this definition does not include findings on the incisors. Thus, our finding that hypomineralization of the maxillary incisors is significantly associated with MIH is particularly important. While incisor hypomineralization is not diagnostic of MIH, based on our results, we conclude that it should raise the suspicion of MIH and lead to an attentive examination of the permanent molars in order to establish timely diagnosis.

## 1. Introduction

Molar incisor hypomineralization (MIH) is a syndrome characterized by demarcated defects of the enamel development that have a systemic origin and that affect one or more of the first permanent molars, with or without involving the permanent incisors [1].

MIH has a multifactorial etiology. Several factors seem to be associated with an increased risk of MIH. These include prematurity, hypoxia-related perinatal problems, as well as a varied range of childhood infections or illnesses (such as urinary tract infection, respiratory tract infections, asthma, or gastrointestinal tract disorders to name only a few) [2].

Furthermore, a predisposition for MIH can also be genetically encoded. For example, an assessment of genome and single-nucleotide polymorphisms (SNPs) in patients and their families has revealed a link between the rs5979395 SNP of the AMELX gene (Xq22) and MIH, with 97% of study participants with MIH carrying the rs5979395*G allele [3].

The characteristics of the hypomineralization defects seen in each MIH case may be influenced by the timing, strength, and duration of these factors during the developmental period of the teeth [2]. A complex interplay between a genetic predisposition and one or more systemic environmental factors would be anticipated to influence teeth in a consistent, time-dependent manner. Nevertheless, when it comes to MIH, any attempt at providing a pathophysiological explanation must account for why teeth developing simultaneously can exhibit differing levels of impact [4].

As described above, several genetic or environmental risk factors for MIH have been characterized, leading to a better understanding of why MIH develops. However, so far it has not been fully understood in whom MIH will develop, as many children experience, for example, repeated episodes of intercurrent illnesses [5,6,7], but not all will go on to develop MIH. This highlights the importance of performing prevalence studies to better explore the burden of MIH in different countries and, potentially, in different settings, as region-specific variations can be expected.

The identification of MIH is based on specific clinical findings, which makes establishing the diagnosis prone to inter-observer bias. Since 2003, the European Academy of Paediatric Dentistry (EAPD) has provided a set of standardized judgement criteria for MIH, making it easier to compare the results across different countries and settings and to generate the most accurate estimates regarding its prevalence [8]. The EAPD recommends that the four first permanent molars and the permanent incisors should be examined for demarcated opacities (with varied size and color, ranging from white to yellow to brown); posteruptive breakdown of affected enamel; and atypical restorations that do not follow the pattern of a cavity but are located in the vestibular, palatal, or lingual aspects, often with opaque enamel at the margins [8]. The incisors should be examined for the existence of vestibular restorations in the absence of dental trauma. In patients with MIH, the first permanent molar may be absent following extraction because of MIH complications; the remaining permanent molars can present atypical restorations surrounded by opaque enamel. We can also notice the failure of eruption altogether of MIH-affected teeth [4,8].

Given the overall polymorphous presentation of MIH, numerous additional disorders could be misdiagnosed for MIH, which is why attentive clinical examination is warranted to ensure a correct differential diagnosis. In addition to identifying the main clinical characteristics, an in-depth anamnesis is crucial for an appropriate diagnosis. This should also focus on the patient’s history in order to determine whether acquired or environmental risk factors for MIH are present.

It is very important to distinguish MIH from other anomalies in the dental structures, such as amelogenesis imperfecta, fluorosis, white spot lesions, or enamel hypoplasia [4]. Compared with MIH, amelogenesis imperfecta is characterized by generalized involvement that can be seen on temporary as well as permanent dentition, with a suggestive family history. In enamel hypoplasia, the lesions generally have regular and smooth borders, whereas in MIH the lesions have a porous and irregular aspect. In fluorosis, the opacities are diffuse in nature, in contrast to the demarcated aspect seen in MIH. White spot lesions are findings suggestive of incipient caries and do not show the specific pattern of MIH, where permanent molars are predominantly affected; white spot lesions occur instead in areas of plaque stagnation, particularly the gingival margin of the tooth, an area which is rarely affected in MIH [4].

Clinical problems in MIH include the rapid loss of enamel leading to posteruptive breakdown, exposing the sub-surface enamel and the dentin on posterior teeth, which is associated with sensitivity to cold air, warm fluids, and, in advanced cases, even to brushing of the affected teeth, which in turn leads to increased susceptibility to dental caries [9]. Chronic pulpal inflammation can occur, associated with sensitivity or even pain, as well as dental fear and anxiety [9]. Eruption difficulties of the molars have been reported, particularly related to the roughness of the enamel. Furthermore, as hypomineralization in MIH can also extend to the incisors, this can lead to aesthetic problems [9]. The prevalence of MIH varies in European countries from 3.6 to 25%. However, these different estimates are very difficult to compare because of differences in study design, diagnostic criteria, age groups, and registration methods. The global mean prevalence, according to a recent analysis, ranges between 13.1% [10] and 14.2% [11].

MIH remains a relevant topic to this day, and real-world prevalence studies are becoming essential in order to quantify the true burden of the disease and to tailor preventive and therapeutic strategies, including modern restorative therapy. A particular emphasis is given to the management of tooth sensitivity or pain, and to preventing the further loss of mineralization. In most cases, without adequate treatment, MIH lesions rapidly progress in severity.

A retrospective study on 414 permanent first molars diagnosed with MIH showed that 43.71% of them had moderate and 26.08% had severe hypomineralization. Dental caries were observed in 71.25% of molars afflicted by MIH. Among these affected molars, 50.72% exhibited uncomplicated carious lesions, while 20.53% presented with complex carious lesions. Notably, 19.64% of these cases warranted consideration for extraction, and ultimately, 9.17% underwent extraction procedures, highlighting the important burden of MIH in the pediatric population [12].

Data about the real-world prevalence of MIH in Romania are scarce [13,14], with only a limited number of studies published so far. The few reports that are available come from dental practice clinics and thus bias results towards either more severe cases or towards a more proactive health-seeking parental behavior. Therefore, more research is needed to quantify the exact burden of MIH in real-world settings in the general pediatric population.

For this reason, we conducted a study with the aim of determining the prevalence of MIH in a population of children attending routine dental consultations in a primary and middle school in Bucharest, Romania.

## 2. Materials and Methods

We performed a cross-sectional study to determine the prevalence of MIH among school children undergoing routine annual dental checkups at one primary and middle school in Bucharest, Romania, from January to June 2023. All children from the first through seventh grades were invited to participate in the study once, as part of the annual dental examination performed routinely by the school dentist. Only those for whom written informed consent was provided by one of the parents or the legal representative before the examination were enrolled.

A single examiner (B.C.) performed all dental checkups after being trained to assess the EADP MIH judgement criteria, which included: well-defined opacities larger than 1 mm in size, lesions of hypomineralization with tissue destruction, atypical restorations with opacities at the periphery, and early absence of permanent molars due to extraction as a result of MIH [8]. All teeth present in the patient’s oral cavity were examined using the standard consultation kit and an external light source. The dental status was noted in the patient file.

MIH can affect one to all four permanent first molars; the incisors may or may not be affected. In order to establish the diagnosis of MIH, at least one permanent molar should be affected. Hypomineralization can also be observed on the tip of the canines or on the second temporary molar. The defect increases in severity as more teeth (molars, incisors) are affected.

Study inclusion criteria:

1. Patients with mixed or permanent dentition.
2. Age between 6 and 15 years old.
3. Participants having at least one permanent molar and one permanent incisor partially or totally erupted, to ensure an accurate examination [8].

Study exclusion criteria:

1. Uncooperative patients.
2. Patients wearing fixed orthodontic appliances.
3. Patients with generalized hypomineralization defects.

The variables that were collected for each study participant included: demographic characteristics such as age and sex, information on the developmental status of the dentition, presence of carious lesions, as well as the presence of demineralization on each of the permanent erupted teeth and on the second deciduous molars.

Ethical approval of the study protocol and the informed consent form was granted by the Research Ethics Committee of the Carol Davila University of Medicine and Pharmacy, Bucharest, Romania, approval number 23159/2023.

Statistical analysis was performed in IBM SPSS Statistics version 25 (IBM Corp., Armonk, NY, USA). For categorical variables, we report absolute and relative frequencies and the results of the Chi square test along with odds ratio (OR) calculation for statistical associations; for non-normally distributed continuous variables we report the median and interquartile range (IQR), along with the results of the Mann–Whitney U non-parametric test. The significance level was set at *p* < 0.05.

## 3. Results

A total of 286 children were enrolled in the study, with a slight predominance (*n* = 157; 54.9%) of males. The age range in the overall cohort was 6–14 years old, and the median age was 10 years old (IQR: 8–11 years). After applying the selection criterion that at least one erupted permanent incisor and one erupted permanent molar are present, 20 children were excluded from the analysis. The remaining evaluable cohort consisted of 266 children, with a similar median age of 10 years old (IQR: 8–11 years); of these, 143 (53.8%) were males.

Overall, 216 children (81.2%) presented mixed dentition, and the remaining 50 children presented permanent dentition. Carious lesions were also identified in 220 of the patients (overall prevalence of 82.7%).

On clinical evaluation, hypomineralizations were present in 40 children (15.0%) in at least one of the evaluated sites. Some of the children presented hypomineralization in two sites (*n* = 16; 6.0%), three sites (*n* = 14; 5.3%), or all four sites (*n* = 7; 2.6%) evaluated. Among these, only two patients who presented hypomineralization at a single site did not meet the criteria for MIH diagnosis. MIH was thus diagnosed in 38 children, resulting in a prevalence of 14.3%.

The frequency of MIH was not statistically different across different patient characteristics, including patient sex (*p* = 0.234), age (*p* = 0.268), presence of mixed vs. permanent dentition (*p* = 0.236), and presence of carious lesions (*p* = 0.502) (Table 1).

Among patients diagnosed with MIH, hypomineralizations were present in *n* = 18 (47.4%) of children on the maxillary first molar, *n* = 35 (92.1%) on the mandibular first molar, *n* = 36 (94.7%) on the maxillary incisor, *n* = 14 (36.8%) on the mandibular incisor, and *n* = 2 (5.3%) on the deciduous second molar.

We next studied the frequency of MIH diagnosis across different sites (i.e., groups of teeth) of hypomineralization and found significant associations between all studied permanent teeth sites (maxillary and mandibular first molar, maxillary and mandibular incisor) (Table 2).

We also assessed the role of the deciduous second molar in the clinical recognition of MIH. In our study group, both patients with hypomineralization on this examined site also had a diagnosis of MIH. However, among those without hypomineralization on the deciduous second molar, 13.6% did have MIH.

## 4. Discussion

To the best of our knowledge, the present study is among the first in Romania that aimed to ascertain the prevalence of MIH among school children during routine dental checkups.

In one other cross-sectional investigation, 277 children were selected at random from three public schools from Bucharest, revealing an MIH prevalence of 9.4% (with a breakdown of 9.03% for boys and 9.83% for girls) [13]. Notably, this prevalence surpasses that reported in an epidemiological study conducted among Romanian school children from the general population. Specifically, this study identified prevalence rates of 5.23%, 3.08%, and 0.71% across three distinct localities within Romania [14].

In a cross-sectional study involving 429 participants, MIH was diagnosed in accordance with EAPD criteria [15]. The prevalence rates were found to be 12.4% for MIH and 5.2% for hypomineralization of the second primary molar. With regard to the distribution of MIH lesions, 5.6% exhibited lesions on both molars and incisors, while the remainder exclusively displayed lesions on molars. Another study involved a substantial cohort of 1138 Syrian school children, revealing a prevalence rate of 39.9% for MIH [16]. Notably, this condition exhibited a higher occurrence among female students, with a prevalence of 41.9%, compared with their male counterparts, who exhibited a prevalence of 37.8%.

In our study, we did not find statistically significant associations between the frequency of MIH and patient characteristics such as biological sex (*p* = 0.234) or age (*p* = 0.268), with the median age being slightly lower, 8 years old, compared with 10 years old in patients with and without MIH, but with overlapping interquartile ranges (8–11 years). This suggests that, by routine clinical evaluation, MIH can be diagnosed relatively soon after the eruption of the permanent molars, which highlights the importance of implementing regular screening in routine clinical practice. School dental offices are ideal for this as they provide coverage of a wide range of children who might not otherwise present to an external dental clinic until markedly visible signs of hypomineralization or persistent or severe symptoms such as dental sensitivity and dental pain occur, which might be too late during the course of disease to allow conservative treatment.

As previously mentioned, data regarding the prevalence of MIH have not been fully explored in Romania [13,14]. Our research discerned a higher prevalence rate (14.3%) than that reported in the few other Romanian studies, in line with data from Italy [17], Sweden [18], Slovenia [19], the Netherlands [20], and the UK [21], but higher than data reported from Bulgaria [22] or Poland [23], while still lower than data from Finland (19.3%) [24] or Sweden (18.4%) [25], as reported in the review by Garg et al. [26].

The results of our current study are important because they provide a real-world estimate from Romania, allowing to a certain extent a comparison with the prevalence reported at the European level.

When studying the frequency of MIH diagnosis across different sites of hypomineralization, we found significant associations that could help orient future diagnostic evaluations. Specifically, the examination of the maxillary incisors was associated with the best odds of predicting a positive diagnosis for MIH, followed in rank by the mandibular first molar as the major diagnostic contributor. This is particularly important since the definition of MIH is the hypomineralization of “one to four permanent first molars” [8]. While MIH is characterized by frequent association with incisor hypomineralization [27], the incisors are not included in the case definition of MIH, albeit they are the most visible of teeth, and changes in their appearance are more easily observed by parents, leading to dental-care-seeking behavior. Our study’s results showed that abnormalities in the aspect of the maxillary and mandibular incisors are statistically important contributors to the routine identification of MIH, even in the absence of a health-seeking behavior bias; these teeth should not be neglected when performing routine dental examinations or MIH screening in children. This is in line with a report from Poland, where half of children with MIH were also shown to present hypomineralizations in permanent incisors [28]. However, our study is the first to show the important difference between the contributing role of maxillary incisors (affected in 94.7% of cases) compared with the mandibular incisors (36.8%).

For the purpose of this study, we also assessed the second deciduous molars. This site is of particular interest because of a potential association that is still being explored between MIH and hypomineralized second primary molars (HSPM). So far, data from the field literature on this topic remain conflicting [15,29]. Our results have confirmed a potential trend for an association between the presence of HSPM and the presence of MIH (*p* = 0.020), which suggests that the deciduous second molar could potentially be an early indicator of a higher risk for MIH. Therefore, routine findings of HSPM should trigger a proactive approach to ensure that early diagnosis of MIH is established at a time when conservative management is still possible. However, if HSPM are not present, this does not exclude the risk of the child eventually presenting MIH (a diagnosis which was present in 13.6% of children without HSPM in our study), and therefore the risk of MIH should not be ruled out based on the absence of HSPM. Furthermore, this finding should also be interpreted with caution due to the small sample size. This is also in line with findings from a study performed in Germany, which identified a lower (3.2%) prevalence of HSPM compared with MIH (9.4%) in rural schools in Central Hesse, and 2.9% HSPM compared with 17.4% MIH in urban schools in Frankfurt on Main [30].

It may be advisable that children with HSPM undergo clinical dental evaluations more regularly around the time when their first permanent molars begin to emerge in order to increase their chances of receiving an early MIH diagnosis. This is particularly important, as the developmental defect seen in dental enamel in MIH is also linked with rapid occurrence and progression of dental caries, which need special attention and warrant early treatment.

According to our findings, 32 out of 38 patients with MIH also have carious lesions (84.2%), and out of 228 patients who do not have MIH, 188 have carious lesions (82.5%). While this is only a preliminary study, it will be interesting to extend the observations in order to determine the frequency of caries in patients with MIH and in those without MIH, depending on the severity of the MIH lesions. The overall high prevalence of dental caries in the pediatric population included in our study can potentially be explained by a complex interplay between a cariogenic, sugar-based diet and the generation of an acidic oral microenvironment, which in turn facilitates the predominance of a cariogenic microbiota [31].

A case–control study of children 7 to 13 years old performed by Grossi et al. compared 130 patients with MIH with 130 controls matched for age, sex, and school. Both between-subject and within-subject analyses found no differences in the frequency of enamel carious lesions but reported that dentine carious lesions were significantly more frequently seen in children with MIH [32]. Furthermore, as part of the within-subject analysis, Grossi et al. also showed that hypomineralized first permanent molars have a higher risk for dental caries compared with the non-hypomineralized molars of the same child [32].

However, data coming from different regions and different studies are not always readily comparable, as they might differ in terms of sample size, type of studied population, number of examiners, or diagnostic criteria applied. Elfrink et al. have highlighted some important recommendations for standardization of studies on MIH, particularly related to the research protocol, calibration sets, and methods applied for diagnosis [33].

Our study comes with a set of limitations, specifically the low sample size for children diagnosed with MIH (*n* = 38), which warrants caution in the interpretation of the large odds ratios obtained in the analysis. Furthermore, data on the severity of MIH were not available at this point as this is a preliminary analysis; this warrants future studies to explore the phenomenon in more depth. Our study also has important strengths, specifically that it is among the first reports highlighting the prevalence of MIH among school children in Romania evaluated in their school environment. This eliminates the bias mentioned above related to the health-seeking behavior of most other studies on MIH performed in clinical settings, and thus provides a better estimate of the burden and prevalence of MIH in real-world settings. As the data come primarily from children attending one urban school in Bucharest, Romania, the findings should therefore not be generalized outside these settings, particularly since a higher prevalence of MIH has been reported in areas of lower socioeconomic status [34] and in rural areas compared with urban areas within the same country [30].

Increasing awareness is very important since various studies regarding the level of knowledge of dentists reported that MIH is a fairly common condition in dental practice. A large percentage of the dentists surveyed have encountered this condition in their clinical practice: 94% in Kuwait [35], 86% in Australia and Chile [36], 81.2% in Iraq [37], and 86.3% in Saudi Arabia [38]. All respondents advocated the need for enhanced clinical training, particularly on the topic of therapeutic approaches in MIH management; this was mentioned as a self-reported need in order to increase dental practitioner confidence in the management of this condition [35,36,37,38].

## 5. Conclusions

In this cross-sectional study, we have reported a prevalence of 14.3% of MIH among school children in real-world settings in Bucharest, Romania. Furthermore, we have noticed that the maxillary incisor and the mandibular first molar represent the most important diagnostic contributors when screening for MIH, highlighting the importance of paying focused attention to these sites during routine dental care in children. Findings of incisor hypomineralization are not diagnostic of MIH; however, our results suggest that these should raise the suspicion of MIH and lead to an attentive examination of the permanent molars in order to establish timely diagnosis.

The presence of hypomineralization on the deciduous second molar appears to be associated with a higher risk for MIH; findings of HSPM should trigger repeated consultations around the time when the first permanent molars erupt. However, the absence of HSPM should not rule out the possibility of MIH occurrence.

In conclusion, MIH appears to be a relatively frequent diagnosis among school children in our setting. It is essential to increase the degree of awareness regarding this condition in order to ensure that diagnosis is established early enough to allow conservative treatment.

## Figures and Tables

**Table 1:** Frequency of molar incisor hypomineralization (MIH) across patient characteristics.

Patient Characteristic	Variable Category	Individuals with MIH (*n* = 38)	Individuals without MIH (*n* = 228)	Total (*n* = 266)	Statistical Analysis
Patient sex	Male	23 (60.5%)	120 (52.6%)	143 (53.8%)	*p* = 0.234
	Female	15 (39.5%)	108 (47.4%)	123 (46.2%)	
Type of dentition	Mixed	33 (86.8%)	183 (80.3%)	216 (81.2%)	*p* = 0.236
	Permanent	5 (13.2%)	45 (19.7%)	50 (18.8%)	
Carious lesions	Present	32 (84.2%)	188 (82.5%)	220 (82.7%)	*p* = 0.502
	Absent	6 (15.8%)	40 (17.5%)	46 (17.3%)	
Age in years	Median (IQR)	8 (8–11)	10 (8–11)	10 (8–11)	*p* = 0.268

Data represent frequency and percentage within MIH category, unless otherwise specified. Categorical variables were compared using the Chi square test. The continuous variable had non-parametric distribution and was compared using the Mann–Whitney U test. The significance level was set as *p* < 0.05.

**Table 2:** Frequency of diagnosis of molar incisor hypomineralization (MIH) across evaluated hypomineralization sites.

Site Assessed	Presence of Hypomineralization	Individuals with MIH (*n* = 38)	Individuals without MIH (*n* = 228)	Total (*n* = 266)	Statistical Analysis
Maxillary first molar	Yes	18 (100%)	0 (0.0%)	18 (6.8%)	OR = 1.9, 95% CI: 1.4–2.6, *p* < 0.001
	No	20 (8.1%)	228 (91.9%)	248 (93.2%)	
Mandibular first molar	Yes	35 (100%)	0 (0.0%)	35 (13.2%)	OR = 12.7, 95% CI: 4.3–37.5, *p* < 0.001
	No	3 (1.3%)	228 (98.7%)	231 (86.8%)	
Maxillary incisor	Yes	36 (94.7%)	2 (5.3%)	38 (14.3%)	OR = 2034, 95% CI: 278–14,899, *p* < 0.001
	No	2 (0.9%)	226 (99.1%)	228 (85.7%)	
Mandibular incisor	Yes	14 (100%)	0 (0.0%)	14 (5.3%)	OR = 1.6, 95% CI: 1.2–2.0, *p* < 0.001
	No	24 (9.5%)	228 (90.5%)	252 (94.7%)	
Deciduous second molar	Yes	2 (100%)	0 (0.0%)	2 (0.8%)	OR = 1.1, 95% CI: 1.0–1.2, *p* = 0.020
	No	36 (13.6%)	228 (85.4%)	264 (99.2%)	

Data represent frequency and percentage within hypomineralization site (i.e., group of teeth). Categorical variables were compared using the Chi square test. The significance level was set at *p* < 0.05. For the purpose of the current analysis, we assessed hypomineralization as present in the examined sites if any of the teeth from that particular group of teeth (site) presented signs of hypomineralization.

## Data Availability

Data are available upon reasonable request from the corresponding author.

## References

[1] Weerheijm K. (2015). The European Academy of Paediatric Dentistry and Molar Incisor Hypomineralisation. Eur. Arch. Paediatr. Dent..

[2] Lygidakis N.A., Garot E., Somani C., Taylor G.D., Rouas P., Wong F.S.L. (2022). Best clinical practice guidance for clinicians dealing with children presenting with molar-incisor-hypomineralisation (MIH): An updated European Academy of Paediatric Dentistry policy document. Eur. Arch. Paediatr. Dent..

[3] Teixeira R.J.P.B., Andrade N.S., Queiroz L.C.C., Mendes F.M., Moura M.S., Moura L.F.A.D., Lima M.D.M. (2018). Exploring the association between genetic and environmental factors and molar incisor hypomineralization: Evidence from a twin study. Int. J. Paediatr. Dent..

[4] Bekes K. (2020). Molar Incisor Hypomineralization: A Clinical Guide to Diagnosis and Treatment.

[5] Çimen B., Aktaş O. (2022). Distribution of bacterial, viral and parasitic gastroenteritis agents in children under 18 years of age in Erzurum, Turkey, 2010–2020. Germs.

[6] Mughini-Gras L., Pijnacker R., Enserink R., Heusinkveld M., van der Hoek W., van Pelt W. (2016). Influenza-like Illness in Households with Children of Preschool Age. Pediatr. Infect. Dis. J..

[7] Paudel G., Amatya N., Saud B., Wagle S., Shrestha V., Adhikari B. (2022). Nasal colonization by potential bacterial pathogens in healthy kindergarten children of Nepal: A prevalence study. Germs.

[8] Weerheijm K.L., Duggal M., Mejàre I., Papagiannoulis L., Koch G., Martens L.C., Hallonsten A.L. (2003). Judgement criteria for molar incisor hypomineralisation (MIH) in epidemiologic studies: A summary of the European meeting on MIH held in Athens, 2003. Eur. Arch. Paediatr. Dent..

[9] Ghanim A., Silva M.J., Elfrink M.E.C., Lygidakis N.A., Mariño R.J., Weerheijm K.L., Manton D.J. (2017). Molar incisor hypomineralisation (MIH) training manual for clinical field surveys and practice. Eur. Arch. Paediatr. Dent..

[10] Schwendicke F., Elhennawy K., Reda S., Bekes K., Manton D.J., Krois J. (2018). Global burden of molar incisor hypomineralization. J. Dent..

[11] Zhao D., Dong B., Yu D., Ren Q., Sun Y. (2018). The prevalence of molar incisor hypomineralization: Evidence from 70 studies. Int. J. Paediatr. Dent..

[12] Luca R., Stanciu I., Tanase M., Zmarandache D., Munteanu A. (2023). First permanent molar extraction in MIH patients OPD 7.12. Eur. Arch. Paediatr. Dent..

[13] Luca R., Farcasiu C., Prelipcean D.D., Stanciu I.A. (2010). MIH in a group of school children from Bucharest, Romania. Abstracts of Papers. Eur. Arch. Paediatr. Dent..

[14] Stanciu I.A., Munteanu A., Luca R., Ionescu E. (2012). Epidemiological study on molar-incisor hypomineralisations in schoolchildren from general population. Med. Evol..

[15] Sidhu N., Wang Y., Barrett E., Casas M. (2020). Prevalence and presentation patterns of enamel hypomineralisation (MIH and HSPM) among paediatric hospital dental patients in Toronto, Canada: A cross-sectional study. Eur. Arch. Paediatr. Dent..

[16] Al-Nerabieah Z., AlKhouli M., Dashash M. (2023). Prevalence and clinical characteristics of molar-incisor hypomineralization in Syrian children: A cross-sectional study. Sci. Rep..

[17] Calderara P.C., Gerthoux P.M., Mocarelli P., Lukinmaa P.L., Tramacere P.L., Alaluusua S. (2005). The prevalence of Molar Incisor Hypomineralisation (MIH) in a group of Italian school children. Eur. J. Paediatr. Dent..

[18] Koch G., Hallonsten A.L., Ludvigsson N., Hansson B.O., Holst A., Ullbro C. (1987). Epidemiologic study of idiopathic enamel hypomineralization in permanent teeth of Swedish children. Community Dent. Oral Epidemiol..

[19] William V., Messer L.B., Burrow M.F. (2006). Molar incisor hypomineralization: Review and recommendations for clinical management. Pediatr. Dent..

[20] Jasulaityte L., Weerheijm K.L., Veerkamp J.S. (2008). Prevalence of molar-incisor-hypomineralisation among children participating in the Dutch National Epidemiological Survey (2003). Eur. Arch. Paediatr. Dent..

[21] Zagdwon A.M., Toumba K.J., Curzon M.E. (2002). The prevalence of developmental enamel defects in permanent molars in a group of English school children. Eur. J. Paediatr. Dent..

[22] Kukleva M.P., Petrova S.G., Kondeva V.K., Nihtyanova T.I. (2008). Molar incisor hypomineralisation in 7-to-14-year old children in Plovdiv, Bulgaria—An epidemiologic study. Folia Med..

[23] Ilczuk-Rypuła D., Zalewska M., Pietraszewska D., Dybek A., Nitecka-Buchta A., Postek-Stefańska L. (2022). Prevalence and Possible Etiological Factors of Molar-Incisor Hypomineralization (MIH) in Population of Silesian Children in Poland: A Pilot Retrospective Cohort Study. Int. J. Environ. Res. Public Health.

[24] Leppaniemi A., Lukinmaa P.L., Alaluusua S. (2001). Nonfluoride hypomineralizations in the permanent first molars and their impact on the treatment need. Caries Res..

[25] Jalevik B., Klingberg G., Barregard L., Noren J.G. (2001). The prevalence of demarcated opacities in permanent first molars in a group of Swedish children. Acta Odontol. Scand..

[26] Garg N., Jain A.K., Saha S., Singh J. (2012). Essentiality of early diagnosis of molar incisor hypomineralization in children and review of its clinical presentation, etiology and management. Int. J. Clin. Pediatr. Dent..

[27] Padavala S., Sukumaran G. (2018). Molar Incisor Hypomineralization and Its Prevalence. Contemp. Clin. Dent..

[28] Glodkowska N., Emerich K. (2019). Molar Incisor Hypomineralization: Prevalence and severity among children from Nothern Poland. Eur. J. Paediatr. Dent..

[29] Estivals J., Fahd C., Baillet J., Rouas P., Manton D.J., Garot E. (2023). The prevalence and characteristics of and the association between MIH and HSPM in South-Western France. Int. J. Paediatr. Dent..

[30] Amend S., Nossol C., Bausback-Schomakers S., Wleklinski C., Scheibelhut C., Pons-Kühnemann J., Frankenberger R., Krämer N. (2021). Prevalence of molar-incisor-hypomineralisation (MIH) among 6-12-year-old children in Central Hesse (Germany). Clin. Oral Investig..

[31] Săndulescu O., Săndulescu M. (2023). Oral biofilms—Pivotal role in understanding microbes and their relevance to the human host. Germs.

[32] Grossi J.A., Cabral R.N., Leal S.C. (2017). Caries Experience in Children with and without Molar-Incisor Hypomineralisation: A Case-Control Study. Caries Res..

[33] Elfrink M.E., Ghanim A., Manton D.J., Weerheijm K.L. (2015). Standardised studies on Molar Incisor Hypomineralisation (MIH) and Hypomineralised Second Primary Molars (HSPM): A need. Eur. Arch. Paediatr. Dent..

[34] Harz D., Catalan Gamonal B., Matute Garcia S., Jeremias F., Martin J., Fresno M.C. Prevalence and severity of molar-incisor hypomineralization, is there an association with socioeconomic status? A cross-sectional study in Chilean schoolchildren. Eur. Arch. Paediatr. Dent..

[35] Alanzi A., Faridoun A., Kavvadia K., Ghanim A. (2018). Dentists’ perception, knowledge, and clinical management of molar-incisor-hypomineralisation in Kuwait: A cross-sectional study. BMC Oral Health.

[36] Gambetta-Tessini K., Mariño R., Ghanim A., Calache H., Manton D.J. (2016). Knowledge, experience and perceptions regarding Molar-Incisor Hypomineralisation (MIH) amongst Australian and Chilean public oral health care practitioners. BMC Oral Health.

[37] Ghanim A., Morgan M., Mariño R., Manton D., Bailey D. (2011). Perception of molar-incisor hypomineralisation (MIH) by Iraqi dental academics. Int. J. Paediatr. Dent..

[38] Silva M.J., Alhowaish L., Ghanim A., Manton D.J. (2016). Knowledge and attitudes regarding molar incisor hypomineralisation amongst Saudi Arabian dental practitioners and dental students. Eur. Arch. Paediatr. Dent..

